# Combination of robot-assisted glove and mirror therapy improves upper limb motor function in subacute stroke patients: a randomized controlled pilot study

**DOI:** 10.3389/fneur.2025.1602896

**Published:** 2025-06-16

**Authors:** Jin Qian, Chong Liang, Run Liu, Jingyi Yu, Tangzhu Yang, Dingqun Bai

**Affiliations:** ^1^Department of Rehabilitation Medicine, Wuchang Hospital Affiliated to Wuhan University of Science and Technology, Wuhan, China; ^2^Key Laboratory of Physical Medicine and Precision Rehabilitation of Chongqing Municipal Health Commission, Department of Rehabilitation Medicine, The First Affiliated Hospital of Chongqing Medical University, Chongqing, China

**Keywords:** mirror therapy, stroke, hemiplegia, upper limb motor function, robot-assisted therapy

## Abstract

**Objective:**

This pilot study aimed to investigate the effects of combining mirror therapy with robot-assisted glove therapy (RMT) on upper limb functional recovery in patients with post-stroke hemiplegia.

**Methods:**

Fifty-two patients with subacute stroke were randomly assigned to three groups mirror therapy (MT) group, robot-assisted therapy (RT) group or RMT group-using a computer-generated randomization table. Patients in all three groups received routine rehabilitation training, MT group received mirror therapy on the basis of this, RT group received rehabilitation robot glove training on the basis of conventional rehabilitation treatment, and the RMT group received rehabilitation robot glove training and mirror therapy at the same time. All interventions lasted for 4 weeks, 5 times a week. Before treatment, 4 weeks after treatment, Fugl-Meyer Assessment-Upper Extremity (FMA-UE), Brunnstrom Hemiplegic Rating Scale, Functional test for the Hemiplegic Upper Extremity–HongKong (FTHUE-HK), Functional Independence Measure (FIM) were used to evaluate the upper limb function and activities of daily living (ADL) of patients.

**Results:**

Compared with baseline, FMA-UE, Brunnstrom upper limb and hand grade and FIM score were observed across all three groups post-intervention (*p* < 0.05). Compared with MT group, FMA-UE (37.61 ± 11.09), Brunnstrom upper limb (4.06 ± 0.87) and hand grades (4.67 ± 1.24) and FIM scores (94.17 ± 9.49) in RMT group were superior after treatment (*p* < 0.05), and the differences were statistically significant.

**Conclusion:**

Mirror therapy combined with rehabilitation robot glove may be an effective treatment method to improve the upper limb function, promote the recovery of motor function and improve the ability of daily living of patients with hemiplegia in subacute stroke.

## 1 Introduction

Stroke, a neurological injury characterized by disrupted cerebral blood flow (1, 2), manifests primarily through two distinct pathogenic mechanisms: ischemic stroke (87% of cases) and hemorrhagic stroke. Ischemic stroke, caused by thrombotic or embolic occlusion of cerebral arteries, triggers excitotoxicity, oxidative stress, and neuroinflammation, leading to neuronal death and functional deficits. Hemorrhagic stroke, caused by cerebral vessel rupture, induces direct tissue damage and complications like intracranial hypertension (3). Recent studies highlight the role of molecular mediators such as progranulin (PGRN), which modulates neuroinflammation and promotes neuronal survival in ischemic stroke (4). The global burden of stroke is staggering, with survivors often facing long-term disabilities, reduced quality of life, and substantial economic costs to healthcare systems (5). In Poland alone, annual direct costs of cardiovascular diseases, including stroke, exceed €4 billion, underscoring the urgency for effective rehabilitation strategies (6).

Globally, 55–75% of surviving patients have upper limb motor dysfunction, and some patients even have secondary problems such as poor flexion and extension of fingers, which seriously affect patients’ activities of daily living (ADL) and quality of life. The upper limb function tends to be fine, and the degree of recovery is affected by various factors. At present, there are many treatments for upper limb dysfunction after stroke, but the efficacy is uneven. Therefore, recovery of upper limb function after stroke has always been a difficult point in rehabilitation (3).

In recent years, mirror therapy (MT), an easy-to-use and cost-effective method, has been widely used in the field of neurological rehabilitation. Studies have reported that MT can activate the mirror neuron system, enhance motor imagination, and thus improve upper limb motor performance (5, 7, 8). However, many stroke patients have poor continuity of attention in the process of MT, resulting in dysregulation of visual and proprioceptive input of patients, affecting the recovery progress of affected limbs, and the abnormal balance of left and right cannot be corrected in time (9). Due to the interference of these limitations with therapeutic efficacy, some studies have found that MT combined with other methods is more effective.

Based on the theory of motor control and learning, robot-assisted therapy (RT), especially gas-driven glove, are beneficial for patients to deepen the limb memory, prevent muscle contracture and improve motor function (10, 11). The pneumatic glove repeatedly inflates and deflates, driving finger joints to perform flexion-extension movements. Gas-driven glove provides patients with high-intensity and repeated exercises, to a certain extent, to make up for the shortcomings of simple MT (8). Because of these advantages, gas-driven glove is gradually being applied as a supplementary means of limb function rehabilitation for stroke patients.

In recent years, combining MT as a central intervention method with other peripheral intervention methods to form a “central-peripheral-central” closed-loop regulation model has become a research focus to improve this technology (12, 13). A previous study found that a combination of robot-assisted glove and mirror therapy (RMT) could promote upper limb movement recovery in stroke patients (14). Studies of robot-assisted arm training after stroke showed that the intervention led to improvements in upper limb function, muscle strength, andADL (15, 16). While robot-assisted therapy is comparable to traditional therapy, combining robot-assisted therapy with other rehabilitation programs has been recognized as a more effective approach to upper limb rehabilitation (17–19). However, these studies have mainly focused on functional recovery in patients in the chronic phase, with fewer studies in patients in the subacute phase.

Therefore, we hypothesized that combining mirror therapy with robotic glove therapy could be used to restore upper limb function in patients with subacute stroke, but its efficacy and safety remain unclear. In this study, we used a randomized controlled approach to compare MT, RT, and RMT differences in upper limb motor function, safety, and feasibility in patients with subacute stroke.

## 2 Methods

### 2.1 Study design and participants

A single-blinded, randomized, controlled clinical trial was conducted from April 2022 to September 2023 in the Department of Rehabilitation Medicine in Wuhan Wuchang Hospital. This trial was approved by the Ethics Committee of Wuhan Wuchang Hospital (No.2022003) and was registered on the Chinese Clinical Trial Registry (ChiCTR 2200057613). Patients after stroke participated in the trial with written informed consents. Treatments involved were performed in accordance with relevant guidelines and regulations.

The inclusion criteria were as follows: first-ever stroke within 6 months; aged 20 to 80 years; unilateral stroke confirmed by CT or MRI; stable condition; upper limb and hand Brunnstrom stages less than grade V. The exclusion criteria were as follows: previous upper limb tendon or neuromuscular injury or other systemic neuromuscular disease; cognitive or language impairment affecting communication; MMSE score less than 24; severe acute and chronic diseases that affect the assessment treatment; Ashworth scale of muscle tension of extensor and flexor fingers more than grade1 plus.

### 2.2 Intervention protocol

#### 2.2.1 Usual rehabilitation treatment and care

**Figure 1:**
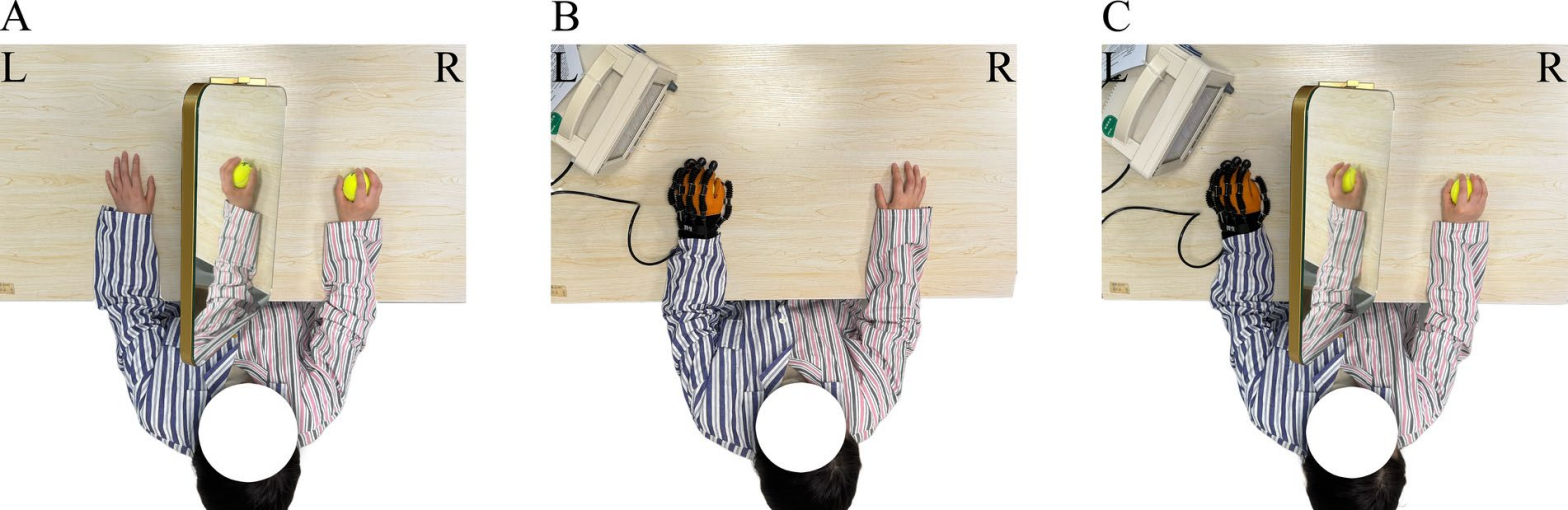
Treatment scenarios. R, unaffected side; L, affected side. **(A)** Mirror therapy setup with a patient observing the reflection of the unaffected limb. **(B)** Patient wearing the Yisheng SY-HR06 robot-assisted glove during training. **(C)** Patient received robot-assisted gloves and mirror therapy simultaneously during training. Patient identities are anonymized, and ethical approval for image use was obtained.

All patients received conventional clinical drug treatment for stroke, as well as comprehensive training for rehabilitative limbs, including normal limb position, physical therapy, occupational therapy, physical therapy modalities (e.g., electrotherapy, thermotherapy) and traditional rehabilitation therapy (20–22). All participants received usual rehabilitation treatment for 30 min/session, 5 sessions/week for 4 consecutive weeks. Participants received MT, RT, or RMT (see Figure 1).

#### 2.2.2 MT protocol

Patients were seated at a table in a comfortable position and places a 60 cm × 50 cm mirror between the two upper limbs in the median sagittal plane, with the reflective side facing the healthy upper limb and the back side facing the affected upper limb. At the same time, the patient is instructed to slightly rotate the torso and head toward the healthy side to observe the reflection of the healthy limb in the mirror, thereby creating the illusion that the affected limb is in motion. During the treatment, the patient is trained to grasp, with the training conducted using a ball. Thirty minutes/session, 5 sessions/week for 4 consecutive weeks.

#### 2.2.3 RT protocol

The Yisheng SY-HR06 rehabilitation robot glove are used for training. The specific steps and parameter settings are as follows: Select the appropriate glove size based on the patient’s hand size, choose the “passive treatment” mode with the healthy hand driving the affected hand, wear the air-driven soft glove on the affected hand, and the patient performs “clench-relax” training while observing the screen. Thirty minutes/session, 5 sessions/week for 4 consecutive weeks.

#### 2.2.4 RMT protocol

Patients were seated at a table in a comfortable position and places a 60 cm × 50 cm therapeutic mirror between the two upper limbs in the median sagittal plane, with the reflective side facing the healthy upper limb and the back facing the affected upper limb. The patient wears an gas-driven soft glove on the affected hand, and is instructed to slightly tilt the torso and head toward the healthy side, gazing at the mirror to perform “clench-release” training. Thirty minutes/session, 5 sessions/week for 4 consecutive weeks.

### 2.3 Outcome measurements

Clinical assessments were used to evaluate the upper limb motor function and self-care abilities of patients in three groups. The clinical assessments included the Fugl-Meyer Assessment for Upper Extremity (FMA-UE), Brunnstrom Scale of Upper Limb and Hand, Functional Test for Hemiplegic Upper Extremity—Hong Kong (FTHUE-HK), and the Functional Independence Measure (FIM). Participants were assessed within 1 week before the intervention (baseline assessment) and after the 20-session intervention (post-assessment). All assessments were conducted by a certified occupational therapist who was unaware of the group assignment of the participant.

#### 2.3.1 FMA-UE

This assessment includes 33 items related to movement, sensation, balance, and joint motion, with a total score of 66 points (23, 24). A higher score indicates less severe upper limb motor dysfunction.

#### 2.3.2 Brunnstrom scale of upper limb and hand

The scale ranges from grade 1 to grade 6, with grade 1 indicating complete inability to move and grade 6 representing normal motor function. A higher grade corresponds to better motor function of the upper limb and hand (24). In this study, grades 1 to 6 were assigned scores from 1 to 6.

#### 2.3.3 FTHUE-HK

It includes 7 levels, level 1 is no reaction, level 7 is the key to unlock the head, control chopsticks (strong), clip (non-strong), the higher the level, the better the function (25).

#### 2.3.4 FIM

The self-care ability of patients was assessed, with a total score of 126 in 18 items. The higher the score, the stronger the ability to perform activities of daily living (26).

### 2.4 Statistical analysis

*A priori* power analysis was conducted using G*Power 3.1. Based on an ANOVA (*α* = 0.05, power = 0.8, effect size *f* = 0.25), the required sample size was 48 (16 per group). Considering a 20% dropout rate, 66 participants were initially recruited.

All tests were executed using the SPSS software version 28 (International Business Machines Corp., Armonk, NY). The Chi-square tests and independent t-tests were used to compare participants’ baseline demographic and clinical characteristics. The measurement data following normal distribution were presented in the form of (mean ± SD). Paired T-test was used for intra-group comparison, one-way analysis of variance (ANOVA) was used for inter-group comparison, and LSD (Least Significant Difference) test was used for further pairwise comparison between groups. Non-parametric test (rank sum test) was used for rank data, Wilcoxon rank sum test was used for intra-group comparison, and Kruskal-Wallis H test was used for inter-group comparison. For all calculations, a significance level at *α* = 0.05 was used.

## 3 Results

### 3.1 Demographic characteristics of three groups

**Figure 2:**
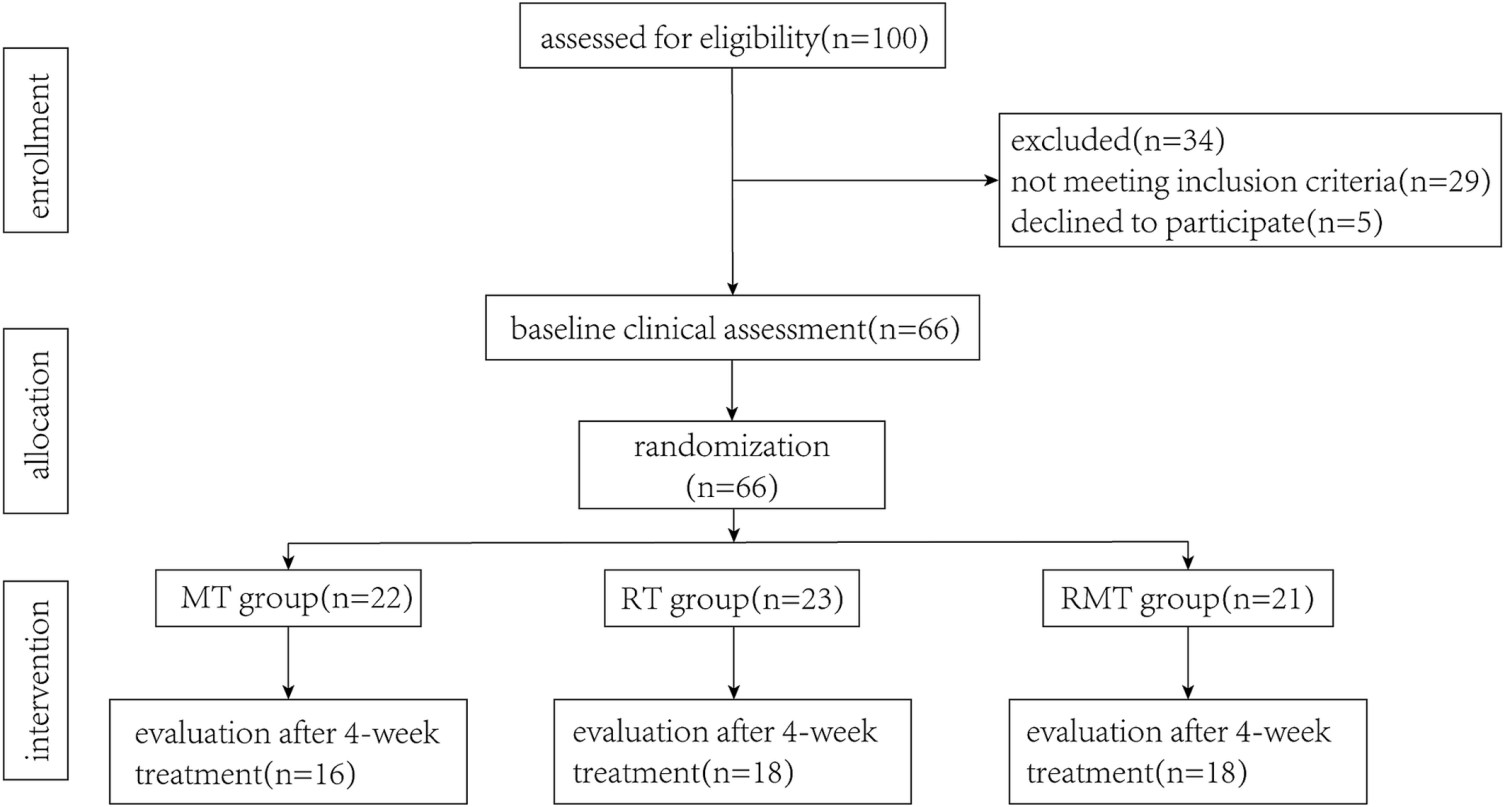
Flow diagram illustrating the flow of participants through each stage of this study.

We screened 100 patients for eligibility. Sixty-six participants met the inclusion criteria and were randomly assigned to three groups. During the intervention period, 14 participants withdrew from the study and were excluded from data analysis (see Figure 2). There were 16 participants in MT group, 18 participants in RT group and 18 participants in RMT group. Descriptive characteristics of participants are presented in Table 1. There were no statistically significant differences in demographic characteristics and clinical presentation between the two groups.

**Table 1:** Demographic characteristics and clinical background of participants.

Variable	MT group	RT group	RMT group	*p*-value
Gender (male/female)	9/7	12/6	14/4	0.41^+^
Affected side (R/L)	6/10	5/13	12/6	0.06^+^
Stroke type	10/6	13/5	12/6	0.83^+^
Age (years), mean ± SD	60.00 ± 7.62	64.28 ± 9.78	62.67 ± 9.95	0.45^*^

MT Group, Mirror therapy; RT Group, Robot-assisted glove therapy; RMT group, Robot-assisted glove combined mirror therapy. ^+^Chi-square test; ^*^One-way ANOVA.

### 3.2 Motor function

**Figure 3:**
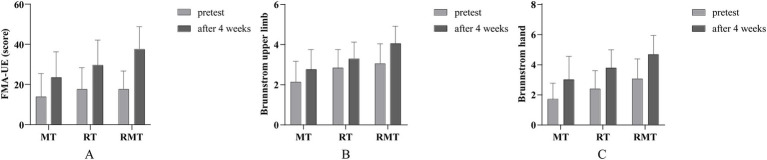
Comparison of motor function improvements across groups. **(A)** Fugl-Meyer Assessment for Upper Extremity (FMA-UE) scores before and after intervention. **(B)** Brunnstrom upper limb grades before and after intervention. **(C)** Brunnstrom hand grades before and after intervention.

The mean and standard deviation for clinical outcome measures are shown in Tables 2, 3 and Figure 3. Results of FMA-UE showed no significant difference among 3 groups before treatment. Compared with before treatment, the above indexes in 3 groups were improved after treatment (*p* < 0.05). Compared with MT group, RMT group showed better performance in FMA-UE after treatment (*p* = 0.006), and the differences were statistically significant.

**Table 2:** Descriptive statistics for clinical outcome measures.

Outcomes	Pretest			After 4 weeks		
	MT	RT	RMT	MT	RT	RMT
FMA-UE	14.00 ± 11.51	17.78 ± 10.54	17.83 ± 9.06	23.63 ± 12.69^ab^	29.78 ± 12.59^a^	37.61 ± 11.09^a^
Brunnstrom-upper	2.13 ± 1.02^**^	2.83 ± 0.92	3.06 ± 1.00	2.75 ± 1.00^ab^	3.28 ± 0.83^ab^	4.06 ± 0.87^a^
Brunnstrom-hand	1.69 ± 1.08^**^	2.39 ± 1.20	3.06 ± 1.30	3.00 ± 1.55^ab^	3.78 ± 1.22^a^	4.67 ± 1.24^a^
FIM	75.75 ± 6.33	79.67 ± 9.00	77.44 ± 7.27	85.69 ± 7.18^ab^	90.28 ± 9.50^a^	94.17 ± 9.49^a^

MT Group, Mirror therapy; RT Group, Robot-assisted glove therapy; RMT group, Robot-assisted glove combined mirror therapy. FMA-UE Fugl-Meyer Assessment for upper extremity, FIM Functional Independence Measure. Data are presented as the mean±SD. **P < 0.05; ^a^*P* < 0.05 = Compared with pretest within the group; ^b^*P* < 0.05 = Compared with the RMT group before and after treatment.

**Table 3:** Descriptive statistics for clinical outcome for FTHUE-HK.

Outcomes	Pretest			After 4 weeks		
	MT	RT	RMT	MT	RT	RMT
Grade 1	7	3	5	3	1	0^a^
Grade 2	6	6	7	3	1^a^	1^a^
Grade 3	2	8	4	7^a^	10^b^	4
Grade 4	1	1	3	3^b^	5	8
Grade 5	0	0	0	0	1	4
Grade 6	0	0	0	0	0	1
Grade 7	0	0	0	0	0	0

MT Group, Mirror therapy; RT Group, Robot-assisted glove therapy; RMT group, Robot-assisted glove combined mirror therapy. FTHUE-HK Functional Test for Hemiplegic Upper Extremity – HongKong Data are presented as n. ^a^*P* < 0.05 = Compared with pretest within the group; ^b^*P* < 0.05 = Compared with the combined group before and after treatment.

As for the results of Brunnstrom upper limb and hand grades, there was no statistically significant interaction between groups before treatment. Subsequent to a four-week treatment period, notable improvements were observed in the Brunnstrom upper limb and hand grades. When compared to the MT group, the RMT group exhibited superior performance post-treatment, with a statistically significant difference (*p* = 0.003).

### 3.3 Self-care

The results of the FIM revealed no statistically significant interaction effect among the three groups (*p* = 0.92). All participants across the three groups demonstrated significant improvements in their independence for daily activities. Notably, the improvements observed in the RMT group were superior when compared to those in the MT group.

## 4 Discussion

In this study, we used upper limb rehabilitation robot glove with merits of high-precision sensing and optimized intelligent algorithm, combined with neuroplasticity induction effect of MT. Our results indicate that compared with the MT group, patients randomized to receive RMT showed a statistically significant average increase of 9.4 points in FMA-UE results. In addition, patients in RMT group showed better improvement in self-care ability (4 point, FIM).

In previous studies, MT based on visual feedback was found to enhance the motor function deficits of the upper limb after stroke (27, 28). Consistent with previous studies, improvements in motor function measured by FMA-UE and FTHUE-HK were observed in all three groups after 4 weeks of intervention. One of the main causes of dysfunction in patients with hemiplegia after stroke is the imbalance of interhemispheric interaction inhibition. Previous studies showed that synchronous movements of bilateral upper limbs generated by rehabilitation robots could reduce the inhibition between cerebral hemispheres (17, 29). Moreover, proprioceptive feedback of the affected side was also conducive to the connection between motor control and primary motor cortex (30), thus promoting sensorimotor integration and showing synergistic gain effect on the activation of sensory and motor areas. In comparison, our results were similar to those of previous studies, with the motor function measured by FMA-UE in RMT group significantly improved compared with MT group alone.

RMT make up for the poor upper limb motor function in the process of image therapy alone, with the support of a previous finding that mirror therapy might effectively improve the Brunnstrom stage and ADL of stroke patients by promoting the neuronal activity (31) of the damaged hemisphere in the motor area and reorganizing cerebral cortex function. In this study, we found that after 4 weeks of intervention, Brunnstrom stage was significantly higher in the 3 groups than before intra treatment, and the improvement in the RMT group was better than that in the MT and RT groups.

Patients with upper limb motor dysfunction after stroke lack participation in ADL (32). Some researchers have combined MT with daily functional activities, and the results show that MT can enhance the motor recovery of the dysfunctional upper limb of stroke patients (13). In this study, we used MT, RT and RMT on stroke patients. After 4 weeks of intervention, the daily function of all patients improved, which was consistent with the results of previous studies on MT (33). In addition, previous studies have shown that MT is more effective than conventional methods in improving the daily function of stroke patients (34). The FIM results of this study showed that when comparing the differences between the two groups, more significant improvements in daily function and self-care ability were observed in the combined group, suggesting that upper limb robotic glove can be used as a supplement to MT alone. This may be related to MT’s activation of motor preparation and brain network separation by inducing image illusion, which promotes motor execution in stroke patients (27, 35).

Several limitations should be acknowledged in this study. Firstly, the RMT group exhibited a higher proportion of right-hemisphere lesions, whereas the MT and RT groups predominantly had left-hemisphere lesions. Although there were no statistically significant differences among the three groups in demography and clinical characteristics of the participants, the hemispherical side of the lesion may affect stroke recovery in upper limb training. Future studies should further investigate whether lateral hemispherical lesions affect the therapeutic efficacy of MT and RT. Secondly, our study did not focus on the differences in the number of grasping times of patients’ hands, since the different degrees of active participation of the patients may affect the results of upper limb function recovery. Thirdly, since we conducted a 4-week intervention without follow-up, the long-term sustained effects of the intervention on stroke patients were unclear. Finally, we used an exoskeleton manipulator for intervention in this study. Studies have shown that exoskeleton robots can be more effective in treating subacute stroke patients with movement disorders (36, 37). Future research should examine whether different movement patterns of exoskeleton robots produce different therapeutic effects on patients with different degrees of movement disorders.

## 5 Conclusion

In summary, the results of this study indicate that, on the basis of conventional rehabilitation treatment, RMT may improve the upper limb function of stroke patients with hemiplegia in the subacute stage, promote the recovery of motor function and improve the ability of daily living activities, and should be considered for promotion and application in the future.

## Data Availability

The original contributions presented in the study are included in the article/supplementary material, further inquiries can be directed to the corresponding authors.
